# Supplementary material for: Understanding parents’ experiences of care for children with medical complexity in England: a qualitative study
Source: BMJ Paediatr Open. 2023 Aug 7;7(1):e002057. doi: 10.1136/bmjpo-2023-002057 (PMC10407344; doi:10.1136/bmjpo-2023-002057)
Supplement: Supplementary data [file bmjpo-2023-002057supp001.pdf]

Research study: Models of care for children with medical complexity

Topic guide for interviews with parents

1. Introducing the interview and consenting process
    - Researcher introduces self
    - Explain/reiterate: confidentiality, length of interview, nature of discussion, withdraw at any time, fine to take breaks
    - Any questions
    - Obtain consent
    - Start recording

2. Finding out about the child and family
    - Exemplar opening scripts: "To start off, please could you tell me a little bit about [name of child]"; "… and the rest of your family?..."

    - Probes:
        - child's favourite activities; nursery/school; personality
        - siblings and ages; family composition
        - name of health condition
        - history of diagnosis

3. Their child's care
    - Exemplar opening script: "And now, please could you tell me a bit about who looks after the healthcare for your child?..."

    - Probes:

        - Which doctors do you see?
        - Do you see any other specialists?
        - Use of medications
        - Does your child have input from therapists e.g. SALT, physio etc
        - Does your child have input from charities?

4. Follow-up questions on target topic areas:
    - Views on how easy it is to access healthcare for their child

- Views on which services should help them look after their child
- Views on how these services could do that e.g. working together, community services flexible options, remote consultations, opening hours etc

CLOSE

- Express thanks and note moving towards end of interview
- Ask if anything else they would like to add

5. Closure:
    - Remind re confidentiality, next steps in research and when findings will be ready
    - Check if any questions
    - Arrange follow-up contact
    - Reiterate appreciation for time and sharing their story.
